# Supplementary material for: Identification of photoperiod-induced specific miRNAs in the adrenal glands of Sunite sheep (Ovis aries)
Source: Front Vet Sci. 2022 Jul 22;9:888207. doi: 10.3389/fvets.2022.888207 (PMC9354845; doi:10.3389/fvets.2022.888207)
Supplement: Supplementary file 1 [file Data_Sheet_1.ZIP › Supplementary file/miRDeep2 file.zip!/miRDeep2 file/Novel_Structure/SPLP42A3/Novel_198.pdf]

Provisional ID : NC_019484.2_39264
Score total : 6.2
Score for star read(s) : 3.9
Score for read counts : -1.3
Score for mfe : 2.1
Score for randfold : 1.6
Score for cons. seed :
Total read count : 9
Mature read count : 7
Loop read count : 0
Star read count : 2

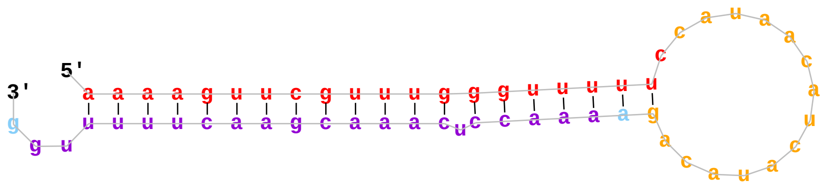

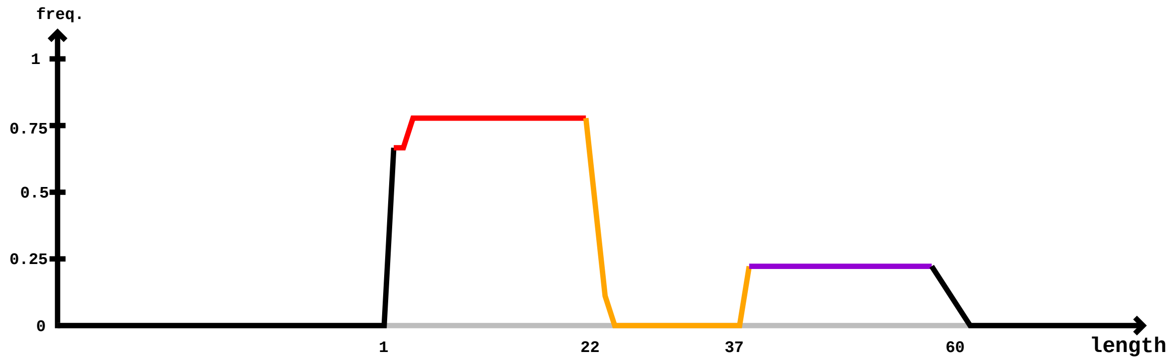


```
                                      Mature                           Star
5'- uuaucagacuggccucuagcuauuggguuggcuaaaaguucguuuggguuuuuccauaacaucauacagaaaaccucaaacgaacuuuuuggccaacccaaugcuacaugg   -3'    obs
    uuaucagacuggccucuagcuauuggguuggcuaaaaguucguuuggguuuuuccauaacaucauacagaaaaccucaaacgaacuuuuuggccaacccaaugcuacaugg          exp
    ............((..((((.((((((((((((((((((((((((((((((((...............))))))).))))))))).))))))))))))))))))))...))   reads   mm   sample
    .................................aaaaguucguuuggguuuuuA.........................................................   5       1    seq
    .................................aaaaguucguuuggguuuuucAa.......................................................   1       1    seq
    ...................................aaguucAuuuggguuuuucc........................................................   1       1    seq
    ......................................................................aaaccucaaaUgaacuuuuug....................   1       1    seq
    ......................................................................aaaccucaaacgaacuuuuug....................   1       0    seq
```
